# Fibre-optic, electronic pH test device compared with current NHS guidance to confirm nasogastric tube placement

**DOI:** 10.1136/bmjnph-2022-000506

**Published:** 2022-12-08

**Authors:** Tracy Earley, Alison Young, Shirley Pringle, Yvonne Clarkson, Alexandra Williams, Rosemary Howell, Marcus Ineson

**Affiliations:** 1 Nutrition, Lancashire Teaching Hospitals NHS Foundation Trust, Preston, UK; 2 Nutrition, Liverpool University Hospitals NHS Foundation Trust (LUHFT), Liverpool, UK; 3 Research, LUHFT, Liverpool, UK; 4 Research, LTHFT, Preston, UK; 5 R Howell Management, Liverpool, UK; 6 NGPOD Global Ltd, Manchester, UK

**Keywords:** COVID-19, Nutritional treatment, Nutrition assessment

## Abstract

The clinical objectives of this prospective, random, convenience series were:

1. Compare a novel fibre-optic pH test device (NGPOD) to gastric aspirate and pH testing for nasogastric tube (NGT) confirmation.

2. Determine if the new device reduces the need for chest radiography (chest X-ray, CXR).

**Methods:**

Recruitment of patients over the age of 18, requiring NGT feeding.

Exclusion criteria: oesophageal gastrointestinal surgery within 3 months; all those with partial or total gastrectomy; bleeding gastric and duodenal ulcers; gastric cancer; those with oesophageal varices; those considered to be inappropriate.

The index test, NGPOD, comprises a fine, flexible fibre-optic sensor passed down the NGT, then connected to an electronic device. A green light indicates pH ≤5.5, and a red light if pH is >5.5.

The reference test is withdrawal of gastric aspirate and testing with universal pH indicator strips then comparison to a colour chart. Second-line testing is establishing NGT position by CXR or subjective clinical assessment (SCA) in intensive care unit (ICU).

**Results:**

The analysed data set contained 174 subjects who had undergone 496 tests, 96 initial and 400 repeat NGT checks.

For all patients, NGPOD can reduce the need for CXR or SCA by 21.2%.

In ICU, NGPOD can reduce the need for CXR or SCA by 24.5%.

When performing initial tests, immediately after tube placement, NGPOD can reduce the need for CXR or SCA in 61% of patients.

With repeat testing, NGPOD can reduce the need to progress to CXR or SCA in 16% of tests.

**Conclusions:**

The objective, yes—no result delivered by NGPOD, eliminates the subjective reading of a pH strip colour change, reducing the subjective element. The index test has the opportunity to reduce risk, improve safety and decrease the numbers of patients requiring X-ray. It, therefore, has the potential to reduce never events associated with NGT misplacement.

**WHAT IS ALREADY KNOWN ON THIS TOPIC**

- There have and continue to be incidents when the misplacement of a nasogastric tube causes severe morbidity and mortality. In the UK, these incidents are referred to as ‘never events’.
- Success in obtaining sufficient aspirate to perform pH testing to check nasogastric tube (NGT) position mean 46%–54% of all NGT insertions require X-ray.
- There is intraoperator and interoperator error in 12%–30% of pH strip results, due to difficulties interpreting the colour change, and timing instructions of the universal indicator strips.

**WHAT THIS STUDY ADDS**

- The index test novel fibre-optic pH test device is able to significantly reduce the number of X-rays, and use of subjective clinical assessment in intensive care unit to determine NGT position.
- The new technology has the opportunity to reduce the numbers of misreported aspirate pH tests and X-rays which cause ‘never events’.

**HOW THIS STUDY MIGHT AFFECT RESEARCH, PRACTICE OR POLICY**

- Introduction of this new technology in to clinical practice has the potential to make NGT position checks more objective by removing the subjective assessment of a colour change.
- Adoption in to clinical practice could transform NGT position checks.
- This clinical study is relevant to both secondary care and home enteral feeding.

## Introduction

Nasogastric tubes (NGTs) are frequently used for the administration of nutrition, hydration and medication. At least one million NGTs are used by the National Health Service (NHS) in the UK annually.^1^ However, there have, and continue to be, incidents where misplacement can cause severe morbidity and mortality. These are referred to as ‘never events’.^2–5^

The National Patient Safety Agency has issued five patient safety alerts (NPSA), and NHS Improvement (NHSI), and additional agencies continue to be concerned with never events.^6–12^

NPSA and NHSI alerts stipulate the first method to confirm NGT placement is pH testing of NGT aspirate. The NGT is considered safe if pH 5.5 or lower is obtained,^7–11^ otherwise chest X-rays are mandated.^7^ Chest X-ray (CXR) is the only approved second-line method of NGT position confirmation when there is no aspirate, or aspirate pH is ≥5.5.

### NGT insertion and aspirate pH testing

Most NGTs in the UK are inserted ‘blind’, as the method does not include any form of visualisation to establish position before use. Hanna *et al* claim 1%–3% of all insertions are misplaced in the lungs, and 19% in the oesophagus and not the stomach.^3^ Tube migration after correct initial placement may be up to 50% in adults.^1^

Success in obtaining aspirate can range from 46%, with 54% of all NGT insertions requiring X-ray confirmation,^13^ and may be up to 87% according to one author.^13–15^ Even when sufficient aspirate is obtained, in a simulated setting, nurses incorrectly read between 12% and 30% of pH strip results.^13^ Misinterpretation of pH 6 as pH 5.5 at a rate of 12%,^13^ shows colour change interpretation to be problematic, which may lead to patient harm.^13 15^ Interpreting a pH 5.5^13^ is a cause of anxiety in 30%–50% of users and may lead to more CXR to check NGT position. The incorrect interpretation of pH strips has been identified as causing 23 out of 95 NGT Never Events from 2011 to 2016.^11 16^

Although CXR is recommended as the second line check for correct NGT placement, and considered by some to be the Gold Standard confirmation method, reporting errors occur.^1 16–18^ Investigation of the 95 never events from 2011 to 2016 found the most common errors (47%) were due to the incorrect interpretation of CXR.^16^

CXR can delay feeding or treatment by several hours,^13 19^ and exposes patients to radiation.^18^ For some patients, there is an inability to obtain aspirate over several days, with repeated exposure to radiation.

Both aspirate pH testing and CXR have limitations.^12^ The BAPEN Special Interest Group recognise that ‘there is a pressing need for an accurate technique to replace pH and CXR, and that where pH is used, automated pH readers could eliminate observer error’.^12^ This study compared a novel pH reader, not requiring aspirate, to the current clinical practice of pH testing of aspirated gastric content and progression to CXR when the first-line test is equivocal.

This study aimed to:

1. Compare the performance of a novel fibre-optic pH test device (NGPOD) to gastric aspirate and pH testing for (NGT) confirmation.
2. Investigate if the new device reduces the need for chest radiography (CXR), or subjective clinical assessment (SCA) in intensive care unit (ICU).

## Method

### Design

This prospective, random, convenience series clinical study compared a novel pH test device to determine NG tube position, to the currently used procedure of testing gastric aspirate with pH universal indicator paper and visual comparison to a colour chart.

### Participants

Participants were adults over the age of 18 years, prescribed enteral feeding by NGT who required verification of NG tube position before the administration of feed, liquid or medication.

The study was structured to recruit sufficient subjects to include 100 first insertion checks (immediately after insertion of NGT) and 500 repeat NGT position checks (before use), with a maximum of 10 tests on each patient.

Exclusion criteria were oesophageal gastrointestinal surgery within 3 months; all those with partial or total gastrectomy; bleeding gastric and duodenal ulcers; gastric cancer; those with oesophageal varices; those considered by their medical team to be inappropriate.

Eligible patients gave informed written consent. When patients lacked capacity relatives, partners or close friends signed a declaration of agreement for participation.

Data collected from participants were: feeding schedule (bolus or continuous regimens); administration route and type of acid-reducing medication (ARM); time since last feed; whether the test was conducted immediately after NGT insertion; or before routine use and any comorbidities.

Three NHS secondary care sites were selected. General wards, ICUs and specialised head and neck wards were used to recruit patients. ICU was selected because feeding regimens are often continuous over 16–24 hours, and it is frequently impossible to gain sufficient aspirate to test, and ARM may increase pH ≥5.5. Specialist head and neck units were included because of the high number of patients requiring NGTs. Fieldwork was conducted between May 2019 and March 2020.

### Index test

**Figure 1:**
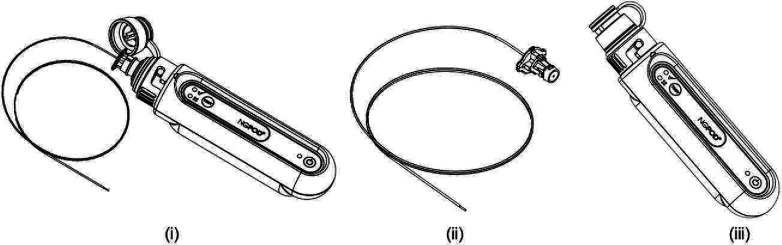
NGPOD system. NGPOD is composed of two elements, a fibre-optic sensor (ii) and a hand-held electronic device (iii). NGPOD, novel fibre-optic pH test device.

Figure 1 shows a diagrammatic representation of the NGPOD system. The distal end of the fibre-optic sensor is coated with a blue hydrophilic pH indicator compound. The sensor is passed through the lumen of the NGT until the tip reaches the interior, distal tip of the NGT. The sensor is connected to the NGPOD device (figure 1) (i) which, when activated, sends a pulse of LED light to the indicator compound at the tip of the sensor. The sensor is a plastic optical fibre, which bends when touched, so is unable to perforate the NGT.

The wavelength distribution of returning light is determined from the output of a photodiode array within the NGPOD device. If a green or yellow wavelength is detected, indicating a pH ≤5.5,^8^ the device will display a green LED tick symbol.^8^ Otherwise the device will display a red LED cross symbol because the pH indicator has not changed from its blue state. This means the tip of the NGT may not be in the stomach, and further NGT position confirmation is required. NGPOD Global has validated the pH detection of the sensor in extensive laboratory testing.

The NGPOD system is CE marked (class 1 electronic device and Sterile Sensors).

### Reference standard

Clinical practice at the time of the research study mandated NGTs to be checked immediately after insertion, and before the administration of clinical feed, liquid or medication. Sufficient gastric contents, 3–5 mL, are aspirated and tested with universal indicator pH strips. A pH of 1–5.5 confirms the NGT to be correctly placed in the stomach.

If the test is pH ≥6, retest after 10–15 min, and if consistently reading pH ≥5.5 confirm NGT position by CXR.

When it is not possible to aspirate stomach contents clinical guidelines recommend:

Repositioning the patient on the side

Flushing the NGT with air

Advancing the NGT by 10 cm and withdrawing slowly reaspirating at 1 cm increments

Aspirate and retest after 15–20 min.

If there continues to be no gastric aspirate, then NGT must be confirmed by CXR.

In ICU, where 16–20 hours feeding regimens are prescribed, ARM medication as well as residual feed remaining in the stomach, may cause the pH of gastric contents to frequently be pH >6.0. Continued exposure to X-ray is harmful to the patient so national guidelines allow the use of local observational procedures to check NGT position.^20^ In this paper, these checks are referred to as SCA.

The index test was always performed as the first test by clinical research nurses (CRNs), trained in the use of NGPOD or nutrition nurses with Good Clinical Practice certification. Immediately after the index test, the reference test was performed by ward nurses, as national guidelines dictate. Index test results were withheld from ward nurses. CRNs collected the data from the aspirate pH testing. In this study, the ward nurses were effectively ‘blind’ to the index tests.

If it was not possible to achieve a result with either NGPOD or aspirate pH testing, according to standard practice,^21^ the nurse was instructed to withdraw or advance the NGT 2 cm; insufflate with air; reposition the patient; wait 10 min before repeat testing.

The same data were collected for the index and reference tests.

NGPOD testing was always performed before withdrawal of aspirate and pH testing. This prevented acidic residue coating the lumen of the NGT, causing accidental contamination of the sensor during insertion.

The study was structured to recruit sufficient subjects to include 100 first insertion checks and 500 repeat NGT position checks, with a maximum of 10 tests on each patient.

### Analysis

McNemar analysis was the preferred analytical approach, comparing paired nominal variables expressed as proportions. Each test occasion is assessed using one or both of the diagnostic tests, with the outcome being expressed as a binary variable: progress to X-ray or SCA (test negative or unsuccessful) vs no progression to X-ray or subjective assessment (test positive).

Two principal populations were defined:

Modified intention to treat (mITT)—either test procedure was carried out (aspirate pH and/or NGPOD). Where one or both tests failed to yield a usable result, it was assumed that the missing test result was negative.

Per-protocol population (PP)—both tests had been successfully carried out and paired results were available.

The results in this paper are presented as an mITT population.

The sample size was determined using the method defined by Machin *et al.* 22

A model, based on a predicted test outcome distribution, produced a requirement for 99 paired data points, with α=0.01 and a β=0.05. The study design, therefore, was considered to have a 95% power to determine consistency between the NGPOD and pH strip testing at a significance level of p=0.01 for the initial placement cohort (proposed 100 patients).

A retrospective recalculation of the required sample size, based on the actual relative test performance, resulted in a requirement for 399 paired results for α=0.01 and β=0.05. As the study actually collected 496 paired results, the power of the study is preserved, despite the difference between modelled and actual test performance.

The study was designed as a comparative test against current practice. It was not designed to specifically determine the sensitivity and specificity of the index test because (1) it would be impractical to definitively determine the outcome (tube position) for every test and (2) any values determined would be specific to clinical setting and test type (initial or repeat) rather than generally applicable.

The primary objective of the study was to assess whether the results obtained with NGPOD are consistent with those derived from conventional pH testing following NG tube aspiration. The clinical outcome used to make this comparison was defined as the requirement to progress to confirmatory X-ray or a SCA (in ICU).

To remove bias in reporting, the analysis of data was performed by an external contractor.

## Results

A total of 176 adults were recruited with 22 subjects removed from the primary analysis set, 15 because of an absence of pH testing and 7 with incomplete data recorded, that is, missing either test result, frequently due to blocked NGT.

Sufficient data existed for analysis on 154 patients undergoing 496 tests. Table 1 presents a summary of this data.

**Table 1:** Number of subjects recruited and results analysed

Characteristic	
Patients	
No	154
Age (mean; SD)	68.0 (15.2)
Gender (% male)	53.2
Setting	
ICU (n; %)	44 (28.6)
Non-ICU (n; %)	110 (71.4)
Tests	
No	496
Initial versus repeat	
Initial (n; %)	96 (19.4)
Subsequent (n; %)	400 (80.6)
Setting	
ICU (n; %)	220 (44.4)
Non-ICU (n; %)	276 (55.6)

ICU, intensive care unit.

There were three types of result obtained over the course of the study:

Tube placement correct

- ‘Green’, NGPOD displayed a Green Tick.
- pH strip results interpreted as pH ≤5.5.

Tube placement incorrect

- ‘Red’, NGPOD displayed a Red Cross.
- pH strip results interpreted as pH >5.5.

Unable to obtain a test result

- ‘No result’ because the NGPOD sensor could not be passed through the NGT, or insufficient contact with an acidic pH.
- pH strip test, ‘no result’ when no aspirate could be obtained for pH testing.

For the mITT population, as well as ICU, initial test and repeat test subgroups, there was a statistically significant reduction in the number of inconclusive tests when using NGPOD, relative to pH aspirate testing.

For all patients in the study, table 2 shows 31.2% of aspirate and pH tests were inconclusive. Use of NGPOD in this population would have reduced the number of inconclusive tests to 24.6%. Inconclusive results drop from 31.2% to 24.6%, representing a 21.2% reduction in the need for CXR or SCA.

**Table 2:** Contingency table analysis for all patients (mITT)

NGPOD (ITT)	pH aspirate (ITT)		Total; N (%)
	pH ≥5.5 or no result	pH ≤5.5 (in stomach)	
Red or no result	86	36	122 (24.6)
Green	69	305	374 (75.4)
Total; N (%)	155 (31.2)	341 (68.7)	496 (100)

Difference between discordant cells: −6.65%, 95% CI −10.66% to −2.65%.

Exact probability (binomial distribution): p=0.0017.

mITT, modified intention to treat; NGPOD, novel fibre-optic pH test device.

Table 3 shows the results for the ICU population. Aspirate pH testing demonstrated a higher number of inconclusive results, 52.3%, compared with 39.5% inconclusive tests with NGPOD. Inconclusive results drop from 52.3% to 39.5%, meaning a 24.5% reduction in the need to progress to CXR or SCA.

**Table 3:** Contingency table analysis for mITT subgroup of subjects in ICU

NGPOD (ITT)	pH aspirate (ITT)		Total; N (%)
	pH ≥5.5 or no result	pH ≤5.5 (in stomach)	
Red or no result	66	21	87 (39.5)
Green	49	84	133 (60.5)
Total; N (%)	115 (52.3)	105 (47.7)	220 (100)

Difference between discordant cells: −12.73%, 95% CI −19.99% to −5.47%.

Exact probability (binomial distribution): p=0.0011.

ICU, intensive care unit; mITT, modified intention to treat; NGPOD, novel fibre-optic pH test device.

Table 4, initial NGT placement checks, when aspirate pH testing returned an 18.8% inconclusive test result, NGPOD would have prevented 11.5% of subjects requiring confirmation by X-ray.

**Table 4:** Contingency table analysis for mITT subgroup undergoing initial testing

NGPOD (ITT)	pH aspirate (ITT)		Total; N (%)
	pH ≥5.5 or no result	pH ≤5.5 (in stomach)	
Red or no result	6	1	7 (7.3)
Green	12	77	89 (92.7)
Total; N (%)	18 (18.8)	78 (81.2)	96 (100)

Difference between discordant cells: −11.46%, 95% CI −18.45% to −4.46%.

Exact probability (binomial distribution): p=0.0034.

mITT, modified intention to treat; NGPOD, novel fibre-optic pH test device.

Inconclusive results drop from 18.8% with aspirate pH testing to 7.3% with the reference test, NGPOD, meaning a 61% reduction in the need to progress to CXR or SCA to determine NGT position.

In table 5, repeat tests, where the NGT is checked before the administration of feed, aspirate pH testing returned 34.2% inconclusive results.

**Table 5:** Contingency table analysis for mITT subgroup undergoing repeat testing

NGPOD (ITT)	pH aspirate (ITT)		Total; N (%)
	pH ≥5.5 or no result	pH ≤5.5 (in stomach)	
Red or no result	80	35	115 (28.7)
Green	57	228	285 (71.2)
Total; N (%)	137 (34.2)	263 (65.8)	400 (100)

Difference between discordant cells: −5.50%, 95% CI −10.17% to −0.83%.

Exact probability (binomial distribution): p=0.0280.

mITT, modified intention to treat; NGPOD, novel fibre-optic pH test device.

Inconclusive results drop from 34.2% with aspirate pH testing, to 28.7% NGPOD, meaning a 16% reduction in the need to progress to CXR or SCA to determine NGT position.

Analysis of results from the PP population showed that the index test performed as well as the reference test in cases where both tests produced a result, with no significant statistical difference between the tests. Analysis of the mITT population, which takes into account the failures to obtain aspirate during the reference test, showed that the index test, NGPOD, results in significantly fewer inconclusive results than the reference test.

Blocked NGTs were responsible for cases where NGPOD and aspirate pH could not provide results. This has been well documented in other publications.^13 14^ Of the 32 occasions where the NGPOD sensor could not be passed, 16 of these also coincided with an inability to obtain aspirate and none of them occurred on initial insertion.

### Adverse events

NGPOD result Green and aspirate pH >5.5—Some tests (n=24) resulted in a Green NGPOD result when the aspirate test was interpreted at pH>5.5. Further investigation by CXR or SCA in ICU, confirmed the NG tube was sited correctly in all these tests.

One discordant result requires a separate comment. The patient, who had undergone extensive oral and facial surgery, required an NGT but was distressed due to being unable to swallow or clear his secretions. The patient did not meet the research study exclusion criteria. The nurse reported the insertion process ‘did not feel right’. The NGPOD test result was a Green (tick), but no aspirate was obtained, and the patient was X-rayed. The X-ray showed that the NGT was in the right main bronchus, and was removed.

## Discussion

This study benefited from a sample size capable of demonstrating clinically significant results. Unfortunately, the well-characterised reference test is known to have inter and intraoperator errors, meaning sensitivity and specificity cannot be determined. In addition, the second line X-ray investigation has inherent inaccuracies, which lead to incorrect interpretations and ‘never events’. This has meant this study is unable to calculate sensitivity and specificity for NGPOD, the usual criteria for measuring the performance of diagnostic tests.

The hands-on diagnostic procedure for testing meant that blinding of results was impossible, as it relied on the ward nurses conducting the aspirate pH part of the procedure. As far as possible, bias in testing was reduced as much as possible, by the CRN team performing the index test.

Despite the above weaknesses, this study has demonstrated the index test is able to generate a result more frequently than aspirate pH testing, because it does not rely on the withdrawal of gastric aspirate. This is a statistically significant finding on general wards, and is particularly evident in ICU. ICU patients are drip-fed over 16–20 hours, which reduces the volume of liquid in the stomach, in addition ARM increases the gastric pH to ≥5.5. If the index test was adopted in ICU it would help to alleviate many subjective observational clinical assessment checks, which concern staff, and have the potential to lead to incorrect determination of NGT position.

The objective yes, no result of the index test demonstrates improved usability over current testing. NGPOD demonstrably reduces the need for repeated attempts at aspirate and pH testing, which cause delays to the administration of nutrition and medication through the NGT. NGPOD has been shown to reduce the number of patients requiring a CXR to confirm NGT position. Removing the need to obtain sufficient gastric aspirate to test is one of the main reasons for the improved performance demonstrated with the index test.

One of the main reasons for no result with NGPOD was due to an inability to pass the fine, flexible sensor through the blockage. The study highlighted the need for high standards of NGT care. Improved NGT care would increase the number of patent tubes and further improve the performance of NGPOD.

The objective, yes, no result delivered by NGPOD, eliminates the subjective reading a pH strip colour change, sometimes performed in poor light conditions and interoperator–intraoperator error. The index test eliminates subjectivity and has the opportunity to reduce risk, improve safety and decrease the numbers of patients requiring X-ray to confirm NGT position, shown to be open to misinterpretation. It, therefore, has the potential to reduce the never events associated with NGT misplacement.

The one discordant result where the NGPOD gave a green tick, but the NGT was found to be in the lung has been extensively investigated clinically, and all equipment thoroughly tested by the manufacturer. No failure of the NGPOD system could be identified as a cause of the green NGPOD result, however, the tube was clearly placed in the lung. Coughing and retching may have caused gastric secretions to be present in the pharynx or top of the trachea, which the NGT encountered and was then transferred to the sensor, accounting for the NGPOD result.

NNNG guidelines recognise that NGT placement in patients who have undergone oropharyngeal surgery, or have significantly altered anatomy, may be contraindicated for blind insertion.^23^

## Conclusion

The novel fibre-optic device, NGPOD, is as accurate as aspirate pH strip testing, and able to deliver a result when it is not possible to obtain aspirate. This is an important factor in the ease of use and ability to deliver a clear and actionable result on general wards and in ICU.

Use of this technology has the potential to increase the number of bedside confirmatory tests for NGT placement, because it is able to deliver a result when it is impossible to withdraw aspirate. It helps to eliminate interoperator–intraoperator error recognised by a BAPEN SIG in 2020. This has a knock-on effect for patients in being able to receive nutrition, hydration or medication on time, and reduce their exposure to X-ray radiation.

Investigations^16^ have found 47% of the most common errors were due to the incorrect interpretation of CXR, so reducing the number of patients requiring CXR to determine NGT position, may go some way to reducing the number of never events.

This study begins to answer the call from The BAPEN SIG in 2020, that there is a pressing need for an accurate bedside device to augment, or replace pH paper and X-ray. Further research and acceptance studies involving greater numbers of subjects and study centres may be necessary before adoption in the NHS.

## Data Availability

Data are available on reasonable request.
